# 

**Published:** 1888-12-22

**Authors:** 


### The Student's Column.

### UNIVERSITY OF LONDON.

### M.B. EXAMINATION FOR HONOURS, 1888.

MEDICINE.

First Class.

May, W. P., B.Sc. (Scholarship and Gold Medal), University College.
Fernando, H. M., B.Sc. (Gold Medal), University College.
*Dean, Henry Percy, B.Sc., University College.
{ Ashworth, Percy, B.Sc., Owens College and Manchr. Royal Infirmary.
{ Parkin, Alfred, Guy's Hospital.
{ Abram, John Hill, University College, Liverpool and London.
{ Pierce, Bedford, St. Bartholomew's Hospital.
{ Wilkie, John, B.Sc., St. Bartholomew's and Bromp. Consumpn. Hospl.

Second Class.

Macevoy, Henry John, B.Sc., St. Thomas's Hospital.
Roberts, John Lloyd, B.A., B.Sc., Guy's Hospital.
Clarke, James Jackson, St. Mary's Hospital.
Bradford, John Rose, D.Sc., University College.
{ Kanthack, A. A., B.A., B.Sc., Liverpool Royal Infiry. and St. Barth's.
{ Starling, Ernest Henry, Guy's Hospital.

Third Class.

O'Reilly, George Hartley, Northampton General Infirmary and King's.
Willoughby, William George, St. Bartholomew's Hospital.
{ Blaxall, Frank Richardson, University College.
{ Canney, Henry Edward Leigh, University College.
{ Williams, Herbert, St. Bartholomew's Hospital.
{ Mair, Ludovic William Darra, St. Bartholomew's Hospital.
{ White, Gilbert Benjamin Mower, University College.

OBSTETRIC MEDICINE.

First Class.

Kanthack, Alfdo. A. (Gold Medal), L'pool. R. Infirmary & St. Barth's.

Second Class.

Sutherland, G. W., B.A., Syd., Univer. Coll., Lond., and Univer., Edin.
Webb, Helen, London School of Medicine and Royal Free Hospital.
{ Dean, Henry Percy, University College.
{ Roberts, John Lloyd, Guy's Hospital.
{ Ashworth, Percy, Owen's College and Manchester Royal Infirmary.
{ Firth, John Lacy, University College.

Third Class.

{ Ashe, Evelyn Oliver, London Hospital.
{ Duncan, Horace, St. Thomas's Hospital and Cambridge.
{ Parkin, Alfred, Guy's Hospital.
Alcock, Samuel King, St. Bartholomew's Hospital.

FORENSIC MEDICINE.

First Class.

Starling, Ernest Henry (Scholarship and Gold Medal), Guy's Hospital.
†Fernando, Hilarion M. (Gold Medal) University College.
†Parkin, Alfred, Guy's Hospital.
*Pierce, Bedford, St. Bartholomew's Hospital.
Canney, Henry Edward Leigh, University College.

Second Class.

Wilkie, John, St. Bartholomew's and Brompton Consumption Hospital.
Gould, John Edwin, University College.
{ Dean, Henry Percy, University College.
{ Kanthack, Alfredo Antunes, Liverpool Royal Infirmary & St. Barth's.
Willoughby, William George, St. Bartholomew's Hospital.
{ Ashe, Evelyn Oliver, London Hospital.
{ Ashworth, Percy, Owen's College and Manchester Royal Infirmary.
{ Melland, Brian, Owen's College and Manchester Royal Infirmary.

Third Class.

Sansom, Harry Arthur, St. Thomas's Hospital.
{ May, William Page, University College.
{ Swayne, Walter Carless, Bristol Medical School and Guy's Hospital.
{ Lyndon, Arnold, St. Bartholomew's Hospital.
{ O'Reilly, George Hartley, Northampton General Infirmary and King's.
{ Duncan, Horace, St. Thomas's Hospital and Cambridge.
{ Sutherland, George Whitefield, Univ. Coll., London, and Univ., Edin.

*Obtained the number of marks qualifying for the University Scholarship.
† Obtained the number of marks qualifying for a Gold Medal.

### M.S. EXAMINATION.

Carless, Albert, King's College.
Dunn, Louis A. (Gold Medal), Guy's Hospital.
Gross, Charles, M.D., Guy's Hospital.

### B.S. EXAMINATION.

First Division.

Ashworth, Percy, B.Sc., Owens College and Manchstr. Royal Infirmary.
Brown, Arthur Thomas, Guy's Hospital.
Crook, Herbert Evelyn, Guy's Hospital.
Cuff, Herbert Edmund, Guy's Hospital.
Dean, Henry Percy, B.Sc., University College.
Goodall, E. Wilberforce, M.D., Guy's Hospital.
Kanthack, Alf. A., B.A., B.Sc., University Coll., L'pool, and St. Bart's.
Moss, Enoch, Guy's Hospital.
Parkin, Alfred, Guy's Hospital.
Starling, Ernest Henry, Guy's Hospital.

Second Division.

Bird, Robert, St. Bartholomew's Hospital.
Brock, Ernest Henry, Guy's Hospital.
Edge, Frederick, B.Sc., Owens College and Manchester Royal Infirmary.
Leech, Priestley, Owens College.
Smith, Guy Bellingham, Guy's Hospital.
Solly, Reginald Vaughan, St. Thomas's Hospital.
Thompson, James Edwin, Owens College.
Tresidder, William Elliot, Guy's Hospital.
Wheeler, James Atkin, Guy's Hospital.

*Obtained the number of marks qualifying for the University Scholarship.
† Obtained the number of marks qualifying for a Gold Medal.
N.B.—The bracket indicates equality of merit.

**To Residents, Secretaries, and Matrons.**

The Editor will be glad to have the Regulations and each Report as issued by Asylums, Hospitals, Convalescent and Nursing Institutions, as well as those of other Charities, and an early copy in duplicate of all Appeals and Festival Notices, etc., addressed to *The Editor, The Lodge, Porchester Square, London, W.* Any superintendent, secretary, matron, or other chief official who regularly sends such information may be registered, on application to the Publisher, to receive free of cost a cover for binding The Hospital in half-yearly volumes.

A Scale of Charges will be found on the first page of the Nursing Supplement.

### Scraps and Gleanings.

Scarlet Fever is on the increase at Derby.

Importation of saccharin is forbidden in France.

Dr. Schofield will shortly publish a medical novel.

Burton Hospital Saturday Fund has reached £787.

Birmingham Hospital Sunday Fund amounted to £4,455.

The Brighton Fancy Ball resulted in £147 for the County Hospital.

The Fulham Vestry has decided to build a mortuary at a cost of £975.

The Kyrle Society hopes to purchase Lambeth Lawn for a public park.

Mr. Val Prinsep is painting the memorial portraits of Dr. Wilson Fox.

The Rev. A. S. Commeline has been elected chaplain of York County Hospital.

The Empress Frederick visited the Throat Hospital, Golden Square, last week.

The Secretary of the West of England Eye Infirmary appeals for Christmas gifts.

Last Monday and Tuesday a bazaar was held at St. Saviour's Hospital, Osnaburgh Street.

Dr. Allinson has lived a fortnight on 2 lbs. of whole meal and 14 quarts of water.

Mr. William Baker, late of St. Leonards, has left £100 to the Middlesex Hospital.

A bazaar was held last week in aid of the Sunderland Infirmary. The Mayor was present.

No less than sixty children have died from measles at Cardiff during the last two months.

M. Pasteur has treated gratuitously 178 British subjects in two years. Death-rate, 3·3 per cent.

Dr. Torbock, of Polruan, has received the Government grant this year for successful vaccination.

Mr. G. Herbert Strutt has been elected president of the Derbyshire Hospital for Sick Children.

The Coventry Hospital has closed its doors to visitors, because of the prevalence of scarlet fever.

Mr. Boutcher, late of Grately, desired to be buried in a wicker coffin, which would not arrest decay.

The Eastbourne Guardians are tired of vaccination prosecutions, and have decided to abandon them.

New York has followed London's example, and started post-graduate lectures at the Medical Colleges.

Dr. Giraud, of Paris, says haschisch is a useful and pleasant drug, and doesn't deserve its bad name.

The Empress of Japan has contributed £16,000 to the Red Cross Society for their hospital at Neno.

Dr. Allinson, of vegetarian reputation, is trying the experiment of living a month on bread and water.

Dr. Field, of St. Mary's, is to have a testimonial when he resigns his post of dean to the Medical College.

An outbreak of typhoid has occurred at Idle, near Bradford. Sixty persons were attacked the first week.

An examination for ten appointments in her Majesty's Indian Medical Service will be held on February 11th.

Only one tender for the erection of a small-pox hospital at Derry was received in answer to local advertisements.

A man certified as dead last week at Lyons, recovered consciousness while in his coffin, and is now alive and well.

Prince George of Wales has been formally received as a Knight of Justice of the Order of St. John of Jerusalem.

The employés of Messrs. Marshall and Snelgrove gave an entertainment in aid of the Middlesex Hospital last Saturday.

Three hundred patients suffering from Beri-Beri were removed in one week from Sumatra to the hospitals at Batavia.

The new Workhouse Infirmary at Birmingham, erected at a cost of £85,000, will be formally opened on January 9th.

Amongst those present at the annual ball in aid of the Nottingham General Hospital was the Duchess of St. Albans.

The Mission to North Sea Fishermen is in future to be granted a certain amount of confiscated smuggled tobacco.

Aberdeen City Hospital is sending out elaborate agreements to be signed by those who desire to participate in its benefits.

The old Empress Augusta of Germany has offered a prize of 10,000 marks (£500) for the best transportable barrack hospital.

The Matron of Radcliffe Infirmary appeals for donations and contributions towards the Christmas entertainment to the patients.

Sir William Turner's lectures at Edinburgh University are daily disturbed by students who sympathise with Sir Morell Mackenzie.

Mr. E. L. Kindersley has offered £100 to the Dorset County Hospital, on the condition that they raise another £200, to clear off the present debt.

Succi, the Italian, has begun a thirty days' fast in the Felipe Theatre at Madrid. He says he is none the worse for his fast at the Barcelona Exhibition.

A bazaar in aid of the Association for Nursing the Sick Poor, and the Creche, was opened at Hove Town Hall on December 13th, by Lady Chichester.

Professor Roser, the eminent surgeon, died at Cassel, on December 17, at the age of 71, from the effects of an apoplectic seizure. The deceased had been for many years one of the leading members of the Medical Faculty at Marburg University.

The tenth festival dinner in aid of the East London Hospital for Children and Dispensary for Women was held at Willis's Rooms, on Thursday, the 20th inst., under the presidency of Mr. Charles A. Prescott, vice-chairman of the board of management.

### Amusements and Relaxation.

### NEW PUZZLE PRIZE SCHEME.

COMMENCED OCTOBER 6th, ENDS DECEMBER 29th, 1888.

On October the 6th a New Prize Scheme was commenced in these columns.

Prizes will be awarded for the best Original Puzzles in Prose or Verse, relating to any of the *Social, Philanthropic, or Political Topics of the day*, and taking the form of Double Acrostics, Anagrams, Square Words, Decapitations, Transpositions, or any other Puzzles which the ingenuity of the competitors may suggest. (*Answers must accompany contributions.*)

*Readers* are requested to send in weekly the Number of Marks they consider each composition to be entitled to, three being the highest score for any one contribution.

Eight Prizes of Standard Books will be given, the choice of the book to be left to the winner.

First, Second, Third, Fourth, and Fifth Prizes for best Original Puzzles £1 1s., 15s., 10 . 6d., 7s. 6d., and 5s. each.

First, Second, and Third Prize for Solutions of 15s., 10 . 6d., and 5s. each.

N.B.—All contributions must reach the office, 38, Old Jewry, E.C., not later than the *First Post* on *Thursday* in each week.

*No Competitor will be allowed to take more than one prize during a quarter.*

### PUZZLES FOR SOLUTION.

### Tenth Week.

No. 25. Musical Puzzle.

The initials to your ears will bring
A tune of Britain's known;
Which some of them refuse to sing,
For reasons of their own.

1. The mother of this earth of ours.
2. A king of fatal fame.
3. The poet of infernal powers.
4. A god with jovial name.
5. A river flowing from the Styx.
6. The Roman chosen few.
7. A place where joy and pleasure mix.
8. The land of fog and dew.
9. A goddess said to be quite blind.
10. A stream with waters black.
11. The fiend disturber of mankind.
12. A boy with wingèd back.
13. The birthplace of a lovely youth.
14. The owners of one eye.
15. Goddess of war, but not of truth.
16. A marvel of the sky.

Morico.

No. 26. Charade.

1. I call and shout to far and near,
To rally round when danger's near.
2. Thousands of feet have trod my way,
Thousands of *this* shall be your pay.
3. Author, who mainly wrote for youth,
In robe of fiction teaching truth.

Our task is o'er, my all receive,
And should you in the midst perceive
Embowered a poet's princess fair,
Grudge not with her my all to share,
And what remains, if she should fly,
That is the season drawing nigh.

Lady Clara.

No. 27. Double Acrostic.

1. Come, guess my first and it will show
An instrument that doctors know.
2. To soar up high my next is able,
By means of both his wings so sable.
3. In ancient days, in Greece, 'tis said,
'Twas here that famous games were played.
4. Without my fourth your Christmas treat
At dinner would be incomplete.
5. Sometimes I'm high, and sometimes low,
Consider when to sea you go.

My finals downward read will spell
A pastime which we all like well;
But if initials you omit,
Impossible you'll render it.

### Marks.

| Names. | No. of Puzzles sent in Dec. 13. | No. of Marks Dec. 13. | Total of Marks. | Names. | No. of Puzzles sent in Dec. 13. | No. of Marks Dec. 13. | Total of Marks. |
|---|---|---|---|---|---|---|---|
| Patience ...... | 3 | 5 | 51 | Hercules...... | — | — | 11 |
| Lady Clara... | 3 | 7 | 58 | Gretta ......... | 3 | 7 | 42 |
| Tinie ......... | 3 | 6 | 48 | Dandy Dick | — | — | 5 |
| Bijou ......... | 3 | 7 | 28 | Boadicea ... | — | — | 7 |
| G. D. ......... | — | — | 15 | Nipper......... | 3 | 6 | 44 |
| Thanet ...... | — | — | 30 | Morico......... | 3 | 8 | 48 |
| Daisy ......... | 1 | 3 | 19 | A. M. E...... | — | — | 3 |
| Salford ...... | — | — | 6 | John Gilpin | — | — | 4 |

### Answers to Eighth Week Puzzles.

No. 21. Double Acrostic.

S qui B
C ucko O
H ypochondri A
O perato R
O rchi D
L ia S

No. 22. Original Anagram.

Chamberlain's Marriage.

### Marks for Solution of Puzzles.

Total Marks.—Ruffles (2), 13; Daisy, 7; Lady Clara (2), 12; Reldas (2), 11; Mater (1), 7; Condorcet, 7; Tinie (1), 12; Gentian (2), 15; Marguerite, 5; Patience, 7; Gretta, 4; Amicus, 7; Morico, 3; Scrooge ([illegible]), 14; Smut, 2; Thanet (2), 17; Jenny Wren 3; Lauriston, 1.

[For Conditions, see last week's The Hospital.]

